# An MRI‐based machine learning radiomics can predict short‐term response to neoadjuvant chemotherapy in patients with cervical squamous cell carcinoma: A multicenter study

**DOI:** 10.1002/cam4.6525

**Published:** 2023-09-29

**Authors:** Zhonghong Xin, Wanying Yan, Yibo Feng, Li Yunzhi, Yaping Zhang, Dawei Wang, Weidao Chen, Jianhong Peng, Cheng Guo, Zixian Chen, Xiaohui Wang, Jun Zhu, Junqiang Lei

**Affiliations:** ^1^ Department of Radiology The First Hospital of Lanzhou University Lanzhou China; ^2^ Infervision Medical Technology Co., Ltd Beijing China; ^3^ Department of Radiology Gansu Provincial Maternity and Child‐care Hospital Lanzhou China; ^4^ Department of Gynecology and Obstetrics The First Hospital of Lanzhou University Lanzhou China; ^5^ Department of Pathology The First Hospital of Lanzhou University Lanzhou China

**Keywords:** cervical squamous cell carcinoma, machine learning, neoadjuvant chemotherapy, radiomics, SVM

## Abstract

**Background and Purpose:**

Neoadjuvant chemotherapy (NACT) has become an essential component of the comprehensive treatment of cervical squamous cell carcinoma (CSCC). However, not all patients respond to chemotherapy due to individual differences in sensitivity and tolerance to chemotherapy drugs. Therefore, accurately predicting the sensitivity of CSCC patients to NACT was vital for individual chemotherapy. This study aims to construct a machine learning radiomics model based on magnetic resonance imaging (MRI) to assess its efficacy in predicting NACT susceptibility among CSCC patients.

**Methods:**

This study included 234 patients with CSCC from two hospitals, who were divided into a training set (*n* = 180), a testing set (*n* = 20), and an external validation set (*n* = 34). Manual radiomic features were extracted from transverse section MRI images, and feature selection was performed using the recursive feature elimination (RFE) method. A prediction model was then generated using three machine learning algorithms, namely logistic regression, random forest, and support vector machines (SVM), for predicting NACT susceptibility. The model's performance was assessed based on the area under the receiver operating characteristic curve (AUC), accuracy, and sensitivity.

**Results:**

The SVM approach achieves the highest scores on both the testing set and the external validation set. In the testing set and external validation set, the AUC of the model was 0.88 and 0.764, and the accuracy was 0.90 and 0.853, the sensitivity was 0.93 and 0.962, respectively.

**Conclusions:**

Machine learning radiomics models based on MRI images have achieved satisfactory performance in predicting the sensitivity of NACT in CSCC patients with high accuracy and robustness, which has great significance for the treatment and personalized medicine of CSCC patients.

## Graphical abstract

We developed a model for predicting susceptibility to neoadjuvant chemotherapy in patients with cervical squamous cell carcinoma using radiomic analysis based on MRI images and independently verified its model generalization ability to predict susceptibility to NACT in another central dataset.

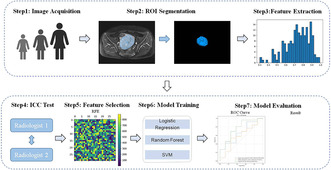

## 1 INTRODUCTION

Cervical cancer (CC) is the most common malignancy in women worldwide and a major cause of cancer death in women, and its incidence rate in younger cases is increasing year by year.^1^, ^2^ The most widely pathological subtype of CC is cervical squamous cell carcinoma (CSCC). It is mainly treated with surgery and radiotherapy, with chemotherapy as a valuable adjunct, according to the International Federation of Gynaecology and Obstetrics (FIGO) guidelines.^3^, ^4^ Concurrent chemoradiotherapy combined with brachytherapy is the standard of care for patients with locally advanced cervical cancer (LACC).^5^ Neoadjuvant chemotherapy (NACT) is an alternative treatment strategy, which can reduce tumor volume, eliminate micrometastases and subclinical lesions, and enable patients who were inoperable to obtain surgical treatment opportunities, and has been widely used in the LACC treatment.^6^, ^7^ In the last few years, NACT has become an essential role in the comprehensive treatment of CC, with continuous improvements in chemotherapy drugs and programs.^8^ However, not all patients respond to chemotherapy due to individual differences in sensitivity and tolerance to chemotherapy drugs, only 30%–50% of patients achieve pathological complete responses.^9^ Therefore, a systematic efficacy evaluation is needed to judge the efficacy of NACT and provide theoretical support for guiding individualized treatment and clinical research.

Currently, efficacy of NACT can be assessed by imaging, pathology, angiogenesis and cell value‐added expression, and serum markers.^10^, ^11^ The pathological evaluation method is more accurate and can judge the prognosis of the patient, but it must be performed after surgery, and this evaluation method cannot be used for patients who cannot undergo surgery.^12^ Evaluating the efficacy of NACT using biomarkers related to angiogenesis and cell proliferation depends on the results of immunohistochemical staining of tumor paraffin sections, which requires clinicians to perform invasive operations to take biopsies again after the initial NACT, or after completing all. After a course of NACT and surgical specimens are taken after surgery, there is a certain risk of delaying treatment in this process.^13^ Serum tumor markers are one of the most used clinical methods for evaluating specific treatment response, but they are insufficient in judging parametrial invasion and lymph node metastasis, and further diagnosis should be combined with imaging examinations.^14^ Imaging examination has the advantages of noninvasive, fast, and convenient, and are more acceptable to both doctors and patients in the clinic.^15^, ^16^

Patients with CC can undergo ultrasound, magnetic resonance imaging (MRI), or CT scans before and after treatment to evaluate the short‐term clinical effectiveness of NACT by comparing changes in tumor size with chemotherapy.^17^, ^18^ Imaging techniques such as ultrasound, MRI, or CT have their own advantages and disadvantages. MRI often has a significant role in the initial stage of the disease for its treatment strategy, treatment planning, and evaluation of tumor response because of good development of endocervical cancer tissue, parauterine invasion, and pelvic wall invasion.^19^, ^20^, ^21^ However, the ability of conventional imaging techniques to predict tumor response is limited, and the radiologist's assessment of imaging results can be subjective.^22^, ^23^ Thus, an objective, noninvasive and highly accurate predictive method is urgently needed to identify patients responding to NACT for CSCC.

In recent years, radiomics is an active research direction, which has attracted more and more attention. It has been widely used in clinical research in tumors diagnosing, and curative effects predicting.^24^, ^25^, ^26^, ^27^ Radiomics aims to quantitatively extract features from diagnostic images and capture the nature of tissues and lesions, as well as to assess the tissue heterogeneity of a patient's tumor.^28^ Biopsy captures heterogeneity in a small subset of tumors, while radiomics captures heterogeneity over the entire tumor volume. Radiomics profiles have been shown to predict response to NACT in patients with CSCC, allowing clinicians to identify patients who may benefit from NACT. Tian et al.^21^ predicted the efficacy of preoperative NACT in patients with LACC by radiological analysis of CT. Arezzo et al.^29^ evaluated lymph node metastases in patients with LACC receiving NACT using machine learning on MRI imaging.^30^ A review of previous studies shows that radiomic features extracted from MRI images have the latent potential to predict the efficacy of NACT.^31^, ^32^, ^33^ However, because of small sample sizes and lack of independent validation, these models are at risk of overfitting and their clinical application is limited. To date, there have been few studies discussing the feasibility of MRI imaging‐based radiomics to predict NACT effectiveness in CSCC patients in a multicenter setting. The limited reproducibility in a multicenter setting remains a key critical bottleneck for the widespread clinical application of radiomics, as demonstrated by extensive studies. Hence, we aim to construct a machine learning radiomics model based on MRI images to predict sensitivity responses to NACT in patients with CSCC and to validate it in a multicenter setting.

## 2 MATERIALS AND METHODS

This retrospective study had the approval of our institution's ethics committee and was exempt from the requirement for informed consent.

### 2.1 Patients

**FIGURE 1:**
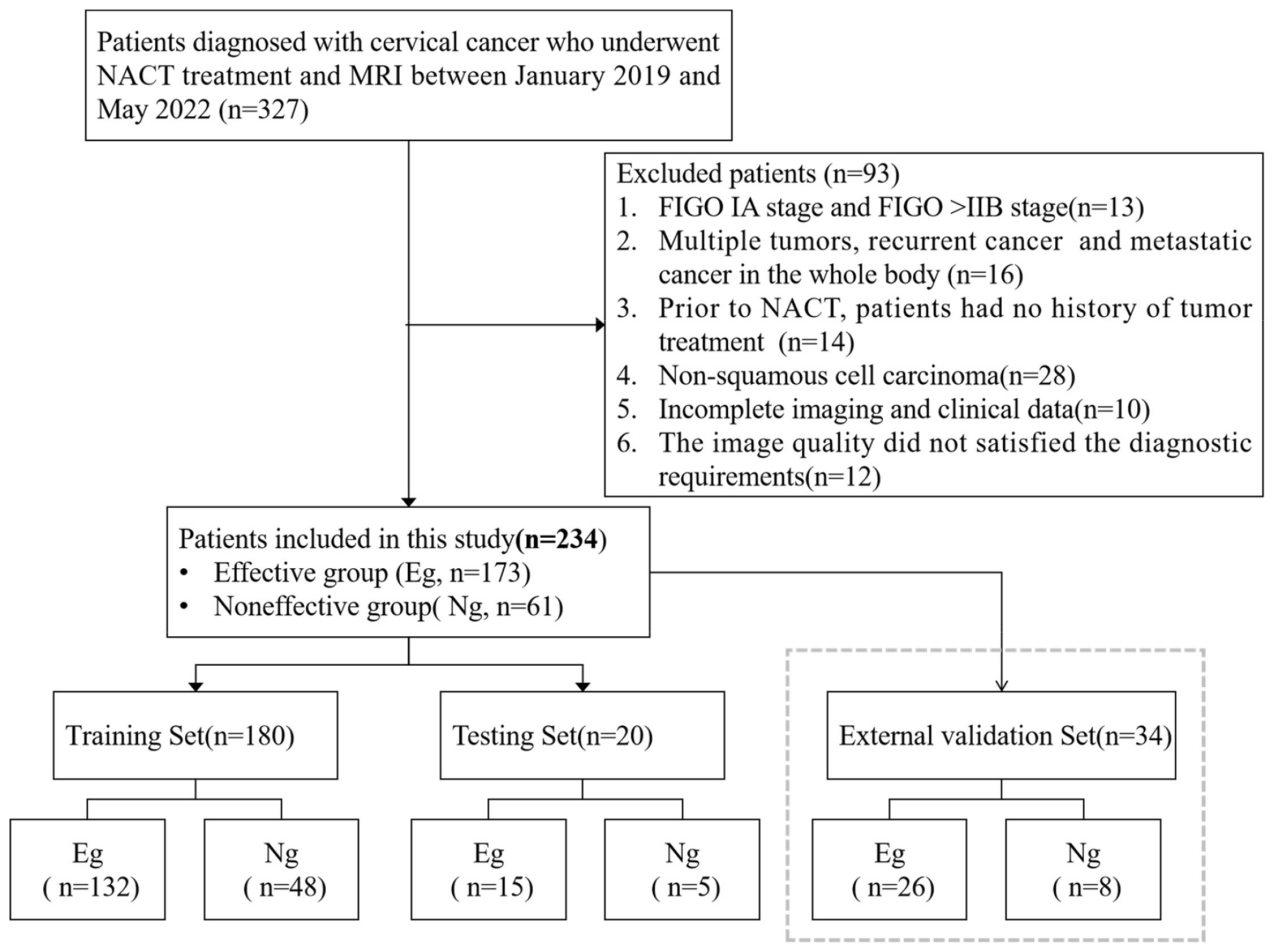
Flow diagram of the patient selection process. It details the number of subjects recruited, excluded, and analyzed in the study.

This study was conducted in the center 1 and the center 2. A total of 234 patients were enrolled from January 2019 to May 2022, including 200 patients from the center 1 and 34 patients from the center 2. All patients had complete clinical data and were confirmed to be CSCC by biopsy pathology, and MRI examination was performed concurrently. The inclusion criteria were as follows: (1) In accordance with the “Obstetrics and Gynecology” diagnostic criteria for CC criteria. (2) Biopsy confirmed CSCC. (3) According to the 2018 FIGO standards determined as stage IB‐IIB CC. (4) No radiotherapy, chemotherapy, and other related treatments were performed before NACT. (5) Pelvic MRI scan was performed before and after NACT treatment. (6) MRI images are clear and meet the diagnostic requirements. The exclusion criteria were as follows: (1) FIGO IA stage and FIGO >IIB stage. (2) Multiple tumors, recurrent tumors, and metastatic tumors. (3) Prior to NACT, patients did not have a history of antitumor therapy. (4) The pathological type was nonsquamous cell carcinoma. (5) Incomplete imaging and clinical data. (6) The quality of the image did not satisfy the diagnostic requirements. The flowchart of the process is as follows (Figure 1).

### 2.2 MRI examination methods

Each patient underwent a routine pelvic MRI sequence scanning within 2 weeks before and 1 month after treatment. The scans were performed on a 3.0 T MR scanner (Ingenia 3.0 T CX, Philips Healthcare; MAGNETOM/SKyra 3.0 T, Siemens Healthcare) in the supine position. To ensure the correct position of the uterus prior to scanning, the bladder was properly inflated. Axial T_2_WI was included in the study. Table 1 shows the protocol details for the MR parameters.

**TABLE 1:** Imaging protocol parameters.

Parameters	T_2_WI
Sequence	MS Multishot TSE
Orientation	Axial
TR/TE (ms)	3391/80
FOV (cm^2^)	220 × 220
Matrix (frequency×phase)	292 × 281
Slice thickness (mm)	3.5
Section gap (mm)	0.35
NSA	1
Bandwidth (Hz/pixel)	213.0
TSE factor	20
Fat suppression	SPAIR
Flip angle (degree)	90
Scan time (min:*sec*)	2.09

Abbreviations: FOV, field of view; NSA, number of signal averaged; SPAIR, spectral attenuation with inversion recovery; TR/TE, repetition time/echo time; TSE, turbo spin echo.

### 2.3 NACT option

All patients were treated with paclitaxel + cisplatin (NACT+RS) regimen, paclitaxel 135‐175 mg/m2d1+ cisplatin 75 mg/m2d1, systemic intravenous infusion, two courses of treatment, each course of 21 days, treatment interval of 2 weeks.

### 2.4 Short‐term efficacy evaluation

The changes of tumor diameter in MRI images were compared and analyzed 2 weeks before and 4 weeks after NACT treatment.

Using RECIST 1.1 criteria, the curative effect was classified as follows: Complete response (CR), the lesion disappeared, partial response (PR), the total length of the lesions decreased by≥30%, stable disease (SD), the total diameter of the lesions decreased but did not reach PR, progressive disease (PD), the total length of the lesions increased by ≥20%, the absolute value of the total length increase must be >5 mm, or new lesions appeared. Accordingly, patients were divided into an effective group (CR + PR) and a noneffective group (SD + PD).^34^, ^35^

At the PACS workstation, a radiologist with more than 10 years of experience in MRI diagnosis independently read the film and measured the largest diameter of the tumor. The measurer was not assigned to the study or involved in the analysis of the results.

### 2.5 Image interpretation by radiologists

**FIGURE 2:**
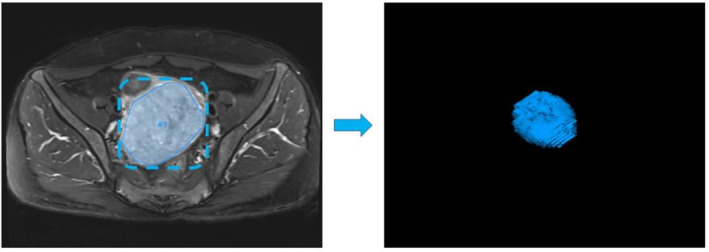
Examples of delineating the region of interest (ROI).

The axial T_2_WI images of all patients before NACT were uploaded to the multimodal scientific research **InferScholar** platform (https://research.infervision.com/v2/) in DICOM format for image annotation. Image segmentation was performed before extracting quantitative radiomics features. A radiologist with over 10 years of diagnostic MRI experience used the research platform to manually delineate each patient's region of interest (ROI) layer by layer on axial T2WI sequences. The ROI was delineated according to the following criteria (1) In combination with multiple sequences, the size, shape, and border of the lesion were carefully observed, and the tumor was delineated as close to the tumor border as possible. (2) ROIs contained liquefaction, necrosis, and cystic degeneration areas in the tumor. (3) ROIs avoid mucus in the cervical canal. A lesion segmentation sample in the dataset is shown in Figure 2.

### 2.6 Radiomics feature selection

**FIGURE 3:**
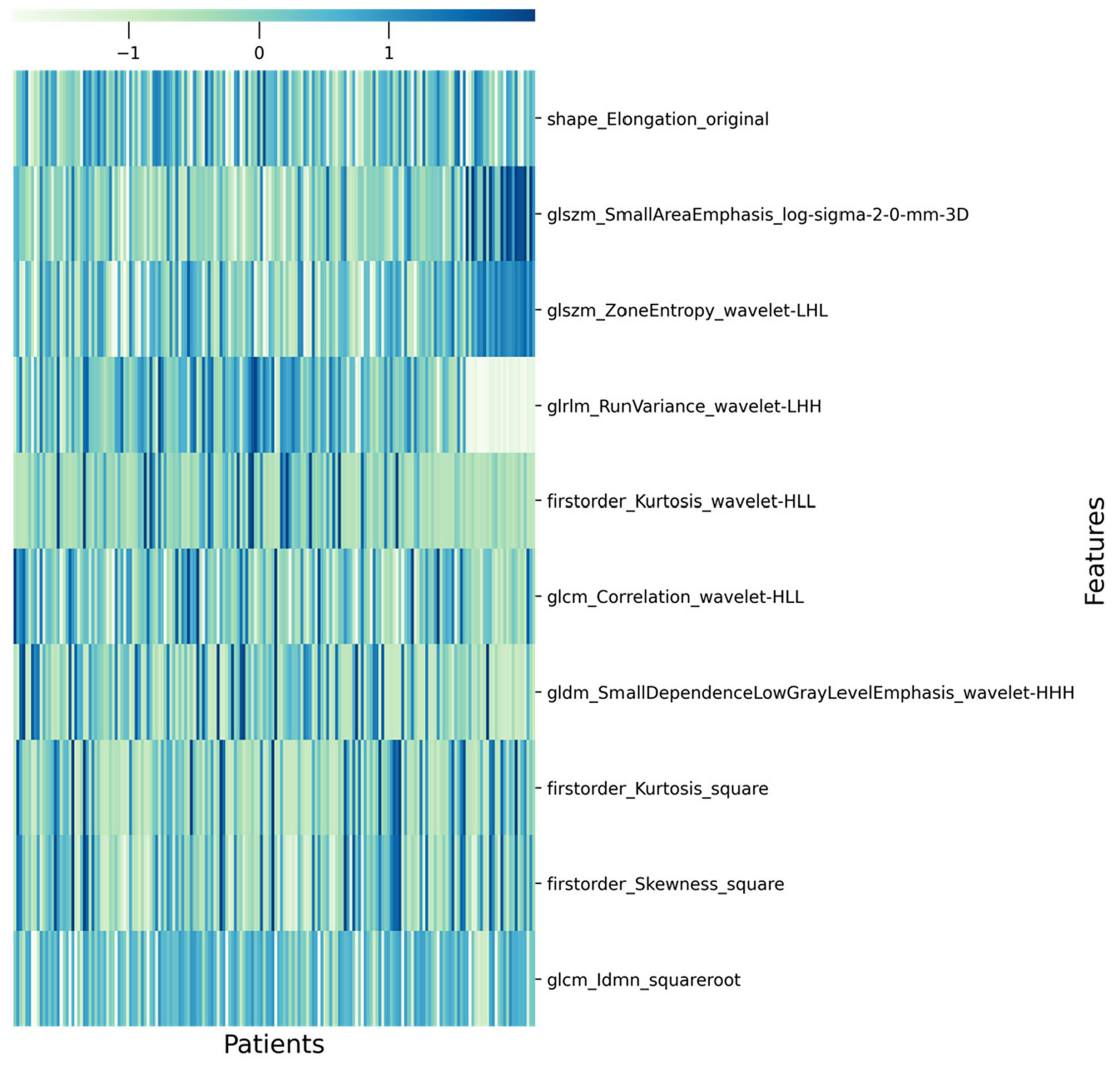
Heat map of radiomic features after feature selection.

In the InferScholar platform, radiomic features such as gray level co‐occurrence matrix were automatically selected from patients' tumor regions. To assess the reproducibility of radiomic feature extraction, intraclass correlation coefficients (ICCs) were used to test the consistency of extracted radiomics in 60 randomly selected patients after 3 months.^36^ The features with an ICC of more than 0.75 were reserved for the selection of the radiomic features. Initially, 1454 radiomic features were extracted from axial T_2_WI images, and 563 radiomic features with ICC >0.75 were preserved by feature elimination at a later stage. The extracted radiomic features include seven categories: first‐order features (*n* = 288), gray level run length matrix (GLRLM) features (*n* = 336), gray level run length matrix (GLRLM) features (*n* = 256), gray level size zone matrix (GLSZM) features (*n* = 256), gray level dependence matrix (GLDM) features (*n* = 224), neighboring gray tone difference matrix (NGTDM) features (*n* = 80), and shape features (*n* = 14).The feature maps for the selected radiomic characteristics are shown in Figure 3.

### 2.7 Radiomics model building and validation

**FIGURE 4:**
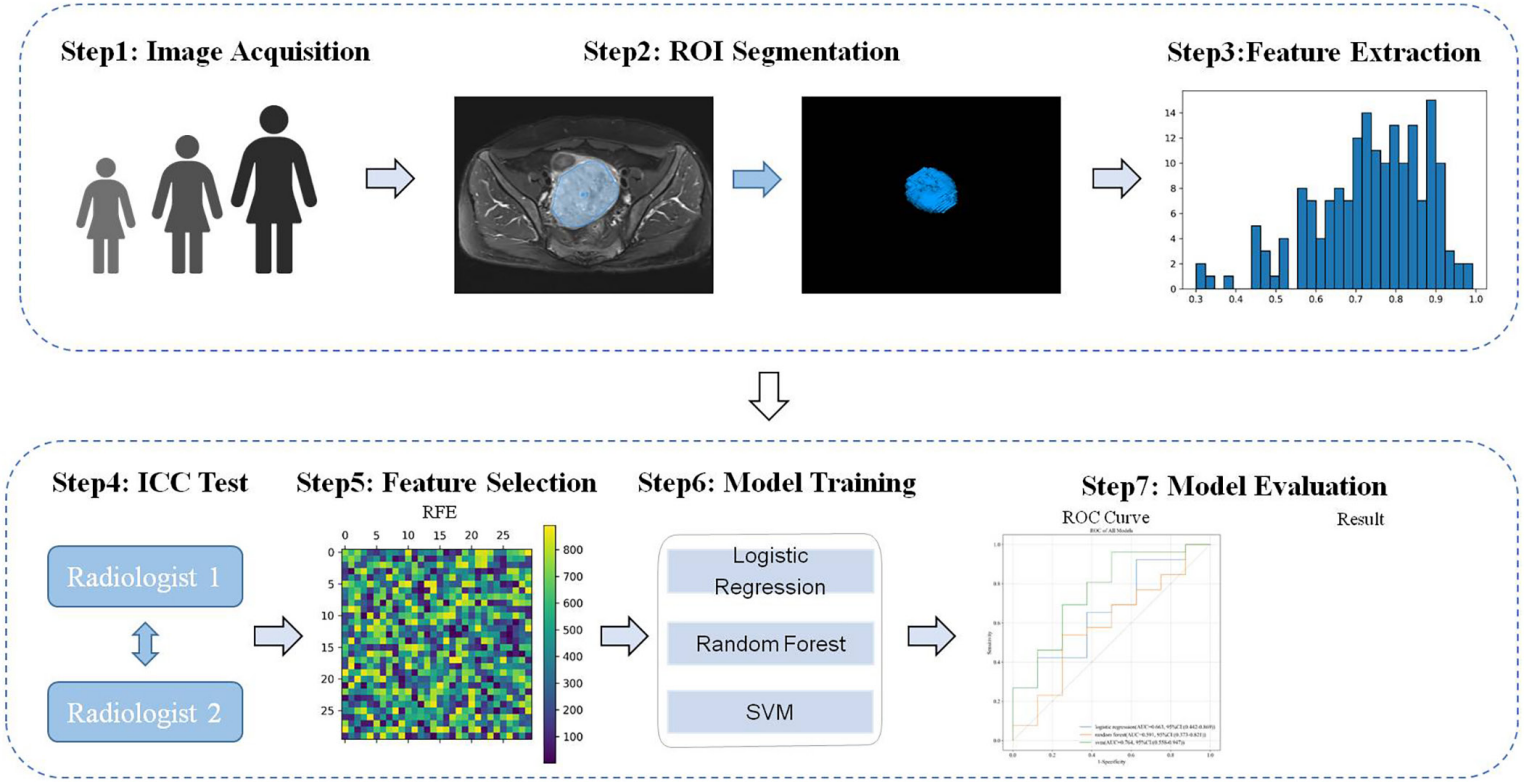
Flowchart of radiomic analysis for predicting the efficacy of neoadjuvant chemotherapy for cervical cancer.

To eliminate redundant radiomics features and improve the model's classification performance of the model, recursive feature elimination (RFE) was used to select the features that contributed the most to the modeling process from the training set. After the feature selection process, a total of 10 features were retained for constructing the radiomics feature set as shown in Table 2. Logistic regression (LR),^37^ random forest (RF),^38^ and support vector machine (SVM)^39^ algorithms were used for the construction of the classification model. In the RF approach, the significance of the features depends on the contribution of each feature to the final decision tree. In the LR method, the importance of features depends on the influence of each feature on the model parameters. In the SVM method, the significance of features is determined which features are important for the classification boundary of the model. We tested the validity of our model on two central datasets and established the model by means of fivefold cross‐validation. The radiomic framework is shown in Figure 4.

**TABLE 2:** Basic information of patients.

	Training set (*n* = 180)		*p* Value	Testing set (*n* = 20)		*p* Value	External set (*n* = 34)		*p* Value
	Eg (*n* = 132)	Ng (*n* = 48)		Eg (*n* = 15)	Ng (*n* = 5)		Eg (*n* = 26)	Ngt (*n* = 8)	
Age (year)	52.43 ± 8.74	51.83 ± 9.82	0.695	49.67 ± 10.11	44.20 ± 7.19	0.282	51.19 ± 7.74	47.25 ± 8.14	0.222
FIGO									
IB	13 (7.22%)	8 (4.44%)	<0.001	1 (5.00%)	1 (5.00%)	0.003	2 (5.88%)	1 (2.94%)	<0.001
IIA	45 (25.00%)	19 (10.56%)		6 (30.00%)	2 (10.00%)		22 (64.70%)	6 (17.65%)	
IIB	74 (41.11%)	21 (11.67%)		8 (40.00%)	2 (10.00%)		2 (5.88%)	1 (2.94%)	
Total	132 (73.33%)	48 (26.67%)		15 (75.00%)	5 (25.00%)		26 (76.47%)	8 (23.53%)	
Diameter of tumor (cm)									
< 4	85 (47.22%)	23 (12.78%)	0.018	5 (25.00%)	4 (20.00%)	0.522	24 (70.59%)	7 (20.59%)	0.0014
4 ~ 6	42 (23.33%)	21 (11.67%)		8 (40.00%)	1 (5.00%)		1 (2.94%)	1 (2.94%)	
> 6	5 (2.78%)	4 (2.22%)		2 (10.00%)	0		1(2.94%)	0	

Abbreviations: Age(y), mean ± SD; Eg, effective group; FIGO, International Federation of Gynecology and Obstetrics; Ng, noneffective group; SD, standard deviation.

### 2.8 Evaluation criteria

Performance of all established models was assessed using ROC analysis, and area under the ROC curve (AUC) was calculated and compared between datasets using the DeLong test. Prediction accuracy, sensitivity, and specificity were also measures.

### 2.9 Statistical analysis

Statistical analysis using SPSS software. Quantitative data were presented as mean ± standard deviation and median (minimum‐maximum). Continuous data were presented as percent. Independent samples *t*‐test and Mann–Whitney U test were used to compare the differences between quantitative variables. Chi‐squared test was used to compare categorical variables. Delong's test was used for the comparison of the difference in AUCs for different models. *p* < 0.05 was considered statistically significant.

## 3 RESULTS

### 3.1 Baseline clinical characteristics

We retrospectively analyzed 327 patients with stage IB‐IIB cervical cancer who underwent radical hysterectomy. Two hundred and thirty‐four patients met eligibility criteria and were enrolled in the study. A total of 180 patients were assigned to the training set, and the mean age of patients was 52 years. Twenty patients were included in the testing set, and the mean age of patients was 48.5 years. Thirty‐four patients were the external validation set, and the mean age of the patients was 51 years. According to RECIST1.1 criteria, 132 patients (73.3%) in the training set, 15 patients (75%) in the testing set, and 26 patients (76.5%) in the external validation set were effective for NACT, respectively. The majority of patients had stage IB (*n* = 26, 11.11%), IIA (*n* = 95, 40.60%), and stage IIB (*n* = 113, 48.29%). Detailed clinical features of all patients are shown in Table 2. In the training set and external validation set, the difference in FIGO stage and tumor size were statistically significant (*p* < 0.001). In the Center 1 dataset, there were 25 patients achieved complete responses and 122 achieved partial complete responses. In the Center 2 dataset, there were four patients achieved complete responses and 22 achieved partial complete responses. The distribution of effective and ineffective NACT treatment in all patients is shown in Table 3.

**TABLE 3:** Distribution of effective and noneffective NACT treatment groups.

		Center 1 (*n* = 200)	Center 2 (*n* = 34)
Effective group	CR	25	4
	PR	122	22
Noneffective group	SD	49	8
	PD	4	0

Abbreviations: CR, complete response; PD, progressive disease; PR, partial response; SD, stable disease.

### 3.2 Feature selection and model results

After feature selection in the training set, 10 features were selected from transverse section images on axial T_2_WI images. The selected radiomic characteristics are shown in Table 4. According to the selected features, three machine learning methods were used to a radiomic model of tumor region based on axial T_2_WI images.

**TABLE 4:** Radiomics features after selection.

Categories	Features
Shape	Elongation original
GLCM	Inverse difference moment normalized square‐root
GLCM	Correlation wavelet‐HLL
First‐order	Skewness square
First‐order	Kurtosis wavelet‐HLL
First‐order	Kurtosis square
GLDM	Small dependence low gray level emphasis wavelet‐HHH
GLRLM	Run variance wavelet‐LHH
GLSZM	Zone entropy wavelet‐LHL
GLSZM	Small area emphasis log‐sigma‐2‐0‐mm‐3D

**FIGURE 5:**
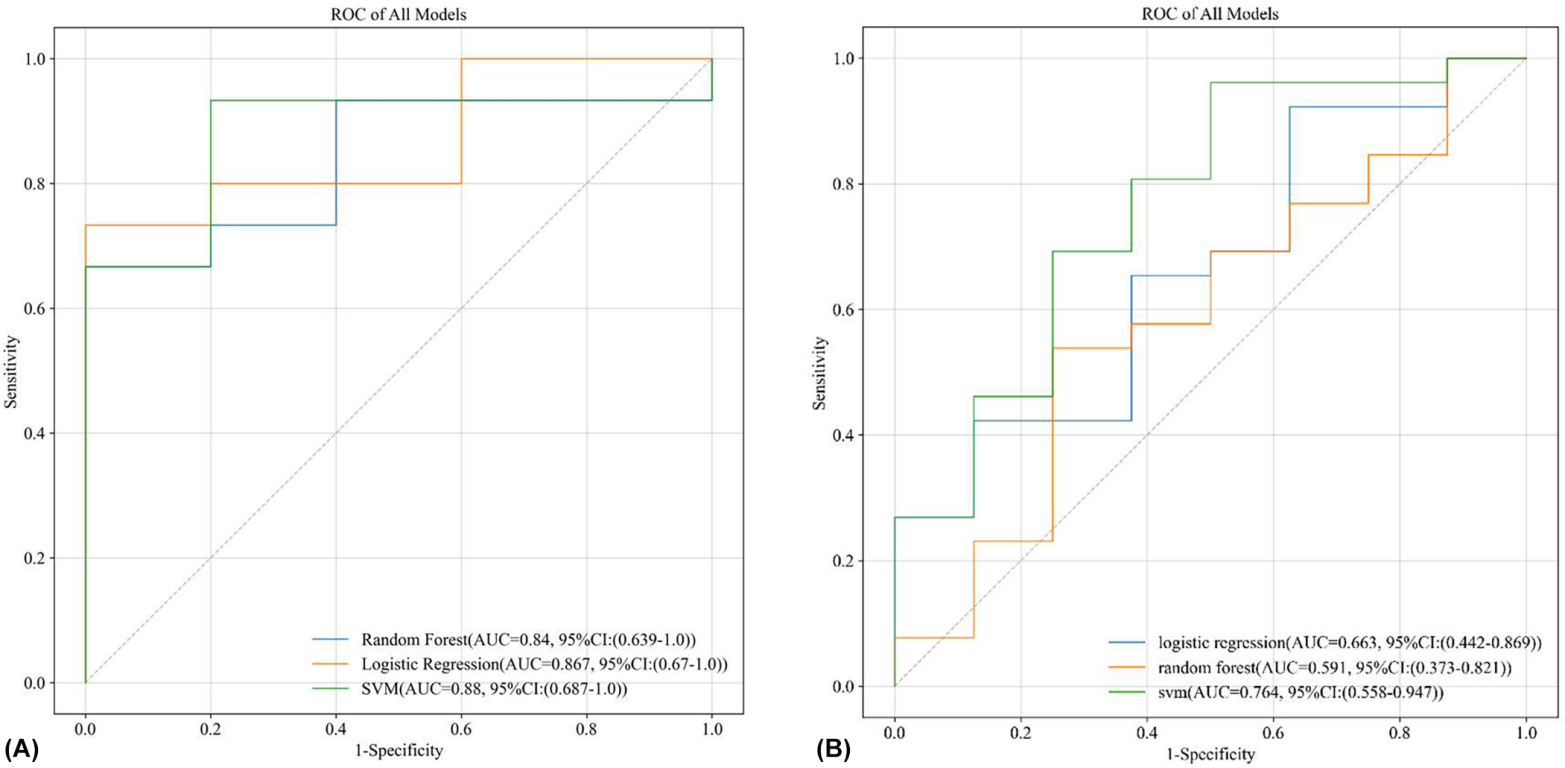
The Receiver operating characteristic (ROC) curves of different machine learning models in the experimental dataset. (A) The testing set radiomic model. (B) The external validation set radiomics model.

Among 563 features with high stability (ICC >0.75), the RFE algorithm was used to screen out the significant features that help to distinguish the efficacy of NACT for CSCC. Finally, the most important features are input into the following modeling process. The prediction ability evaluation of three machine learning classification methods under the sensitivity prediction model based on NACT for CSCC patients is shown in Table 4 and Table 5. The Table 5 is the evaluation of the testing set, and the Table 6 is the evaluation of the external validation set. To further evaluate the model performance of different methods, the Figure 5A,B provides ROC curves of the above models in the testing set and external validation set, respectively.

**TABLE 5:** Predictive performance of the model in the testing set.

Model	Accuracy (95% CI)	Sensitivity (95% CI)	Precision (95% CI)	F1 (95% CI)	AUC (95% CI)
Random Forest	0.75 (0.55–0.95)	0.667 (0.438–0.901)	**1** (1.0–1.0)	0.8 (0.609–0.948)	0.84 (0.639–1.0)
Logistic Regression	0.80 (0.60–0.95)	0.733 (0.50–0.938)	**1** (1.0–1.0)	0.846 (0.667–0.968)	0.867 (0.67–1.0)
SVM	**0.90** (0.75–1.0)	0.933 (0.778–1.0)	0.933 (0.769–1.0)	0.933 (0.827–1.0)	**0.88** (0.687–1.0)

Bold indicates the highest result in the comparison of the results of the three machine learning methods.

Abbreviations: AUC, area under the receiver operating characteristic curve; CI, confidence interval.

**TABLE 6:** Predictive performance of the model in the external validation set.

Model	Accuracy (95% CI)	Sensitivity (95% CI)	Precision (95% CI)	F1 (95% CI)	AUC (95% CI)
Logistic regression	0.794 (0.647–0.913)	0.923 (0.808–1.0)	0.828 (0.679–0.962)	0.873 (0.76–0.954)	0.663 (0.442–0.869)
Random forest	0.588 (0.441–0.765)	0.538 (0.346–0.724)	**0.875** (0.684–1.0)	0.667 (0.486–0.816)	0.591 (0.373–0.821)
Svm	**0.853** (0.735–0.971)	0.962 (0.875–1.0)	0.862 (0.724–0.968)	0.909 (0.818–0.982)	0.764 (0.558–0.947)

Bold indicates the highest result in the comparison of the results of the three machine learning methods.

In both the testing set and the external validation set, we clearly found that the SVM approach performed better, with an AUC score of 0.88 (95% CI: 0.687–1.0) for the testing set and 0.764 (95% CI: 0.558–0.947) for the external validation set. The sensitivity prediction model of NACT for CSCC patients based on axial T_2_WI images and radiomics can also achieve high accuracy in evaluating the efficacy response. In the SVM method, the accuracy of the testing set is 0.90 (95% CI: 0.75–1.0), and the accuracy of the external validation set is 0.853 (95% CI: 0.735–0.971). Among them, in the test set, logistic regression and random forest have higher accuracy than the SVM method, and their scores can reach 1.0 (1.0 vs. 0.933 (95% CI: 0.769–1.0)). In the external validation set, the random forest method has higher accuracy than the other two machine learning methods, and its score is 0.875 (95% CI: 0.684–1.0).

## 4 DISCUSSION

In this study, we constructed a machine learning radiological model based on MRI images for predicting susceptibility responses to NACT in CSCC patients and independently validated the model's ability to predict NACT efficacy in another central dataset. Experimentally, SVM showed better performance in predicting sensitive responses to NACT, providing useful and valuable information for future treatment of CSCC patients.

The tumors of CSCC patients are clinically heterogeneous, and not all patients benefit from NACT. Histopathological examination is still the gold standard for assessing response in clinical practice. However, it can only be performed after surgery, which delays the timely adjustment of treatment. Thus, accurate prediction of treatment response is very important. Predicting the response of CSCC patients to NACT has been increasingly reported. Some studies have performed radiomic analysis on T_1_WI and DWI sequences, but rarely on axial T_2_WI images analysis. According to research, axial T_2_WI images can accurately determine whether the parastatal tissue is invaded, especially for stage I CSCC, the invasion of the cervical matrix should be observed. CSCC lesions on axial T_2_WI images appear as medium signal masses, and low signal fibrous matrix truncation is a diagnostic criterion for lesions. DWI is mainly used to observe the dispersion and the motions for water molecules in living tissues through two‐dimensional images, and mildly detect the motion of water molecules in human tissues through signals of diffusion sensitive gradient tissues in response, to judge the pathological conditions of human body. Compared with DWI, axial T_2_WI images have advantages in contrast and clarity, allowing more accurate delineation of the lesion area.^40^ Therefore, this study attempted to analyze the axial T_2_WI images to predict the response of CSCC patients to NACT.

The field of radiomics research has recently received increasing attention, aiming to quantitatively extract features from diagnostic images, capture the properties of tissues and lesions, and forming a high dimensional feature space that can be developed. Radiomic features combined with machine learning methods to build potential predictive and prognostic models to assist in guiding clinical diagnosis. Machine learning methods include a variety of algorithms. Song et al. preliminary study successfully used pretreatment CT radiomics to predict the pathological response of locally advanced gastric cancer after NACT.^41^ Tian et al.^29^ extracted radiomic features from 277 patients receiving NACT for LACC, and the results indicated that the radiomics features could be used as effective predictors to help patients perform risk stratification and improve the selection of NACT. Marco et al.^42^ evaluated pathological complete responses to NACT in breast cancer using a machine learning radiomics approach in dynamic enhanced MRI. However, the clinical relevance of these studies is limited due to the relatively small sample size and the lack of validation in a multicenter collaborative setting.

The model established in our study shows good performance in predicting the sensitive response of NACT and verifying the multicenter environment. In the SVM method, the AUC score of the test set was 0.88 (0.687–1.0), accuracy, sensitivity, precision, and F1 score were 0.90 (0.75–1.0), 0.933 (0.778–1.0), and 0.933 (0.769–1.0), respectively. In the external validation set, the prediction ability of the model also showed good performance, with an AUC score of 0.764 (0.558–0.947), accuracy, sensitivity, accuracy, and F1 score of 0.853 (0.735–0.971), 0.962 (0.875–1.0), 0.862 (0.724–0.968), and 0.909 (0.818–0.982), respectively. In addition, compared with the three machine learning methods, LR and RF also reached the highest accuracy of 1 (1.0–1.0) in the testing set, and RF reached the highest accuracy of 0.875 (0.684–1.0) in the external validation set. Therefore, the predicting model we constructed based on the sensitivity response of CSCC patients to NACT is feasible to guide the clinical treatment plan and implement personalized treatment.

It is worth mentioning that this study has some limitations. Firstly, although data from different centers were selected, due to retrospective data and different distributions, inherent bias was inevitable. Therefore, we need to further collect patient cases from different regions and construct a prospective study to demonstrate the generality and clinical value of this model. Secondly, the study only used MRI axial T_2_WI images, MRI has a variety of sequences, DWI can observe the degree of CC penetration, sagittal T_2_WI images can show cervical anatomy, the detection of lesions is very sensitive. Therefore, in the future, we can analyze the response of different sequences and multiple sequences fusion to NACT sensitivity in CSCC patients. Finally, we only used axial T_2_WI images and did not include clinical risk factors affecting patients' NACT. Some studies have shown that clinical information has a high influence on model prediction.^43^ Therefore, clinical factor analysis is worthy of further study. Future studies combining imaging and clinical data to create a joint prediction model may provide more microinformation that can be tested on larger patient populations and in different centers, benefiting more patients. In conclusion, we propose an MRI‐based machine learning radiomics model that can noninvasively predict NACT‐sensitive responses in CSCC patients and has the potential to provide guidance to clinicians in the decision‐making process for tailoring treatment to CSCC patients.

## AUTHOR CONTRIBUTIONS

**Zhonghong Xin:** Data curation (equal); investigation (equal); supervision (equal); writing – original draft (supporting); writing – review and editing (equal). **Wanying Yan:** Formal analysis (equal); project administration (equal); visualization (equal); writing – original draft (equal); writing – review and editing (equal). **Yibo Feng:** Methodology (equal); software (equal). **Yunzhi Li:** Data curation (supporting). **Yaping Zhang:** Data curation (supporting). **Dawei Wang:** Writing – review and editing (supporting). **Weidao Chen:** Software (supporting). **Jianhong Peng:** Data curation (supporting). **Cheng Guo:** Data curation (supporting). **Zixian Chen:** Data curation (supporting). **Xiaohui Wang:** Data curation (supporting). **Jun Zhu:** Data curation (supporting). **junqiang lei:** Resources (supporting); supervision (equal).

## FUNDING INFORMATION

This study was supported by Department of Radiology, the First Hospital of Lanzhou University (center 1), Intelligent Imaging Medical Engineering Research Center of Gansu Province, Accurate Image Collaborative Innovation International Science and Technology Cooperation Base of Gansu Province, Department of Radiology, Gansu Provincial Maternity and Child‐care Hospital (center 2), and the Science and Technology Program of Gansu Province is greatly appreciated (20JR10RA684).

## CONFLICT OF INTEREST STATEMENT

The authors have no conflict of interest.

## Supporting information


Appendix S1

## Data Availability

Data sharing is not applicable to this article as no new data were created or analyzed in this study.
